# Implications of High Temperature and Elevated CO_2_ on Flowering Time in Plants

**DOI:** 10.3389/fpls.2016.00913

**Published:** 2016-06-27

**Authors:** S. V. Krishna Jagadish, Rajeev N. Bahuguna, Maduraimuthu Djanaguiraman, Rico Gamuyao, P. V. Vara Prasad, Peter Q. Craufurd

**Affiliations:** ^1^International Rice Research InstituteMetro Manila, Philippines; ^2^Department of Agronomy, Kansas State UniversityManhattan, KS, USA; ^3^International Maize and Wheat Improvement Centre (CIMMYT)Nairobi, Kenya

**Keywords:** climate change, flowering time, flowering regulation, high temperature, elevated CO_2_, tissue temperature

## Abstract

Flowering is a crucial determinant for plant reproductive success and seed-set. Increasing temperature and elevated carbon-dioxide (e[CO_2_]) are key climate change factors that could affect plant fitness and flowering related events. Addressing the effect of these environmental factors on flowering events such as time of day of anthesis (TOA) and flowering time (duration from germination till flowering) is critical to understand the adaptation of plants/crops to changing climate and is the major aim of this review. Increasing ambient temperature is the major climatic factor that advances flowering time in crops and other plants, with a modest effect of e[CO_2_]_._Integrated environmental stimuli such as photoperiod, temperature and e[CO_2_] regulating flowering time is discussed. The critical role of plant tissue temperature influencing TOA is highlighted and crop models need to substitute ambient air temperature with canopy or floral tissue temperature to improve predictions. A complex signaling network of flowering regulation with change in ambient temperature involving different transcription factors (*PIF4, PIF5*), flowering suppressors (*HvODDSOC2, SVP, FLC*) and autonomous pathway (*FCA, FVE*) genes, mainly from *Arabidopsis*, provides a promising avenue to improve our understanding of the dynamics of flowering time under changing climate. Elevated CO_2_ mediated changes in tissue sugar status and a direct [CO_2_]-driven regulatory pathway involving a key flowering gene, *MOTHER OF FT AND TFL1* (*MFT*), are emerging evidence for the role of e[CO_2_] in flowering time regulation.

## Introduction

The Intergovernmental Panel on Climate Change Reports (e.g., IPCC, 2007, 2013) document evidence of increasing carbon-dioxide concentrations (e[CO_2_]) and other greenhouse gases leading to a higher frequency of extreme climate events such as heat waves and drought events. The impact of these climate events has already been documented on agricultural crop production, natural species diversity and distribution, and other ecosystem services such as flowering time, pollination etc. (Dale et al., 2001; Doney et al., 2012). Given that temperature is a major determinant of the timing and duration of key developmental phases, including flowering (Bahuguna and Jagadish, 2015), and [CO_2_] a major determinant of plant growth (Craufurd and Wheeler, 2009), climate change is likely to have significant impacts on key flowering processes.

Flowering time is defined here as the duration starting from seed germination till appearance of the first floral bud, open flower or anthesis (external appearance of anthers). Flowering time marks the visible transition from the vegetative to reproductive phase and is important because: (i) the duration from emergence to flowering determines the reproductive competency of a plant; (ii) the timing of flowering *per se* relative to the occurrence of abiotic and biotic constraints is critical for successful seed-set and propagation; (iii) the timing of flowering in ecosystems or plant communities may affect individual species “fitness” in relation to competition with other species; (iv) the synchrony of pollination with insects in ecosystems and hence the distribution of species may be affected. All the above functions can be affected by climate change; thus flowering time is one of the major factors determining the adaptation of plants to changing climate. Environmental factors such as photoperiod and temperature that control the timing of the first bud or flowering from a whole-plant physiological perspective has been described in detail by Craufurd and Wheeler (2009). In general, historical records of flowering time, herbaria and aerobiological documents on pollen data indicate advancement in flowering time in perennials (Crimmins et al., 2011; Hulme, 2011). However, shifts in flowering time among annuals, such as wheat (*Triticum aestivum*), which may be influenced by changes in crop management practices such as variable sowing dates, have to be interpreted with caution (Craufurd and Wheeler, 2009). The above is also true under conditions wherein crop breeding efforts are targeted toward earlier or later flowering to increase cultivar adaptability to environments faced with terminal or end of season drought stress.

A number of physiological processes that occur during anthesis, such as pollination, pollen germination and fertilization, are highly sensitive to extremes of temperature. The time of day of anthesis (TOA), which is defined as the appearance of the first open flower or anthesis on any particular flowering day, can also vary and provide an escape route to overcome the damage induced by high temperature. For example, in tropical and sub-tropical rice environments ambient air temperature gradually increases from early morning through solar noon and late afternoon, and decreases thereafter. Pollination occurs at or just prior to anthesis and fertilization is generally completed within a few minutes or hours after pollination (Cho, 1956; Kakani et al., 2005). High-temperature effects on processes from pollination through fertilization can be minimized by exploiting variation in the TOA, i.e., by selecting species or cultivars that flower earlier in the morning when temperatures are cooler. Some wild rice varieties have shown extremely large variation in the TOA, with *Oryza alta* flowering after 2100 h at night and *O. officinalis* as early as 0600 h in the morning (Sheehy et al., 2007). This variation, particularly from *O. officinalis*, has been exploited to develop rice varieties that flower a few hours earlier (toward dawn) in a day when temperatures are cooler, leading to enhanced seed-set even with exposure to early-afternoon hotter temperature (Ishimaru et al., 2010; Hirabayashi et al., 2015). Hence, some of the negative impacts of increasing temperature on the flowering events that affect seed-set (i.e., pollination, pollen germination and fertilization) can potentially be avoided by identifying and exploiting such unique flowering patterns, at least in annual grasses. This review aims to highlight ambient temperature and e[CO_2_] regulation on TOA and flowering time and implications for crop improvement under future climate.

## Canopy and tissue microclimate affects TOA

Plants require a certain amount of heat units (thermal-time), synonymous with growing degree-days, to reach and progress to the next developmental stage. Provided the temperature is below a critical threshold, plants achieve these requirements earlier in warmer than cooler temperatures, thus increasing the rate of development (Craufurd and Wheeler, 2009). Many plant or crop models using cardinal temperatures (i.e., base, optimum, maximum temperature) and growing degree-days to drive development do not adequately account for the impact of high temperatures or short-term extremely high temperature stress events (but see Asseng et al., 2015). In addition, for species or cultivars sensitive to photoperiod, a greater understanding of the interactions between photoperiod and temperature above the optimum temperature is still required to improve existing crop/flowering models, especially for predicting responses to warmer and more extreme high-temperature events.

Research on high-temperature responses of mostly annual crop species such as rice (*Oryza sativa* L.) or sorghum (*Sorghum bicolor*), using controlled-environment conditions, has provided extensive knowledge on different high-temperature tolerance and escape mechanisms (Ishimaru et al., 2010; Jagadish et al., 2010; Jain et al., 2010; Talukder et al., 2013). Simulation models estimating temperature impacts on plants and crops have generally used standard meteorological air temperatures. However, canopy or floral tissue temperature can be considerably lower or higher than air temperature depending on soil water status and the microclimate surrounding sensitive plant organs (Yoshimoto et al., 2011; Guo et al., 2013; Julia and Dingkuhn, 2013). Significant variation in rice panicle temperature, from 9.5°C below to 2°C above ambient air temperature, was recorded with a common set of rice cultivars tested across contrasting environments in Senegal, France and the Philippines (Julia and Dingkuhn, 2013). Likewise, a 6°C lower rice panicle temperature in semi-arid climates of Australia (Matsui et al., 2007) and 4°C higher panicle temperature under humid conditions in China (Tian et al., 2010) and other rice-growing locations has been documented (Yoshimoto et al., 2011). The above provides strong evidence for large variation in air temperature and tissue (flower or spike or panicle) temperature, which can strongly influence flowering dynamics such as TOA (Julia and Dingkuhn, 2012). Interestingly, these differences in tissue temperature can be attributed largely to variation in the prevailing atmospheric relative humidity and hence demand for water mediated through vapor pressure deficit (VPD). This relationship strongly correlates with TOA and ultimately percent spikelet sterility (Shimono et al., 2005; Julia and Dingkuhn, 2013). Nonetheless, some effects attributed to changes in air temperature can be equally accounted for by water-deficit stress in crop models (such as maize in APSIM) that represent physiological processes (Lobell et al., 2013). This is because warmer temperatures increase VPD and demand for water that can lead to water-deficit stress.

There is also variation among species in their ability to regulate tissue temperature in floral organs. The presence of rigid epidermal pores on rice spikelets leads to lower flexibility in altering panicle temperature (Takahashi et al., 2008). The presence of active stomata on wheat glumes and awns (Steinmeyer et al., 2013) and buds of *Brassica* spp. (Guo et al., 2013) has been shown to actively moderate reproductive tissue temperatures when exposed to both high-temperature and water-deficit stress. Interestingly, water loss from detached leaves and buds of *Brassica* spp. indicated immediate closure of stomata on the leaf while the bud retained stomatal activity, extending evaporative cooling and leading to a delay in increasing bud temperature under drought stress (Guo et al., 2013). Canopy temperature depression (CTD) is being used as a measure of water-deficit stress avoidance facilitated by deeper roots in wheat (Lopes and Reynolds, 2010), differential leaf wax content for variation in reflecting radiation (Mahmud et al., 2016) or as a result of genetic variation in stomatal conductance in response to variation in VPD (Reynolds et al., 2007; Jagadish et al., 2011). The same concept has been recently extended to ear temperature depression (ETD) in wheat (Steinmeyer et al., 2013). Accounting for the variation in air and tissue (ear/floral) temperature can increase the precision of estimating TOA and seed-set in hotter environments. Hence, crop models parameterized with air temperature alone in environments varying in relative humidity or VPD do not predict responses to high temperatures well (for example, CSM-CROPSIM-CERES-Wheat, White et al., 2011; Asseng et al., 2015, for a comparison of wheat models). Thus, microclimate data that includes canopy and floral tissue temperature (including soil water potential while dealing with upland cereals) is proposed as the appropriate approach to fine-tune crop improvement traits such as TOA, in crop models as demonstrated recently for flooded rice (Julia and Dingkuhn, 2012, 2013; Yoshimoto et al., 2011).

Although the increase in global mean surface temperature is well documented, recent reports indicate a more rapid increase in minimum night temperature compared to maximum day temperature at the global (Vose et al., 2005), regional (Welch et al., 2010), and farm/field (Peng et al., 2004) level, reducing the day-night temperature amplitude (Christensen et al., 2007). This phenomena of warmer nights and warmer mean temperatures, leads to earlier TOA in cereals such as rice (Kobayashi et al., 2010; Julia and Dingkuhn, 2013). For example, variation in the mean minimum air temperature during the 7 days preceding anthesis was negatively correlated (*R* = −0.85) with the variation in time of day at peak anthesis (maximum number of open spikelets; Julia and Dingkuhn, 2012). In contrast, Shi et al. (2013) found no significant change in the flowering patterns in rice exposed to higher night temperatures between panicle initiation and flowering in a semi-controlled field experiment. Hence, more research on the effects of temperature amplitude on TOA, flowering pattern and other reproductive processes is needed.

## Flowering time

### Impact of increasing temperature

**Figure 1:**
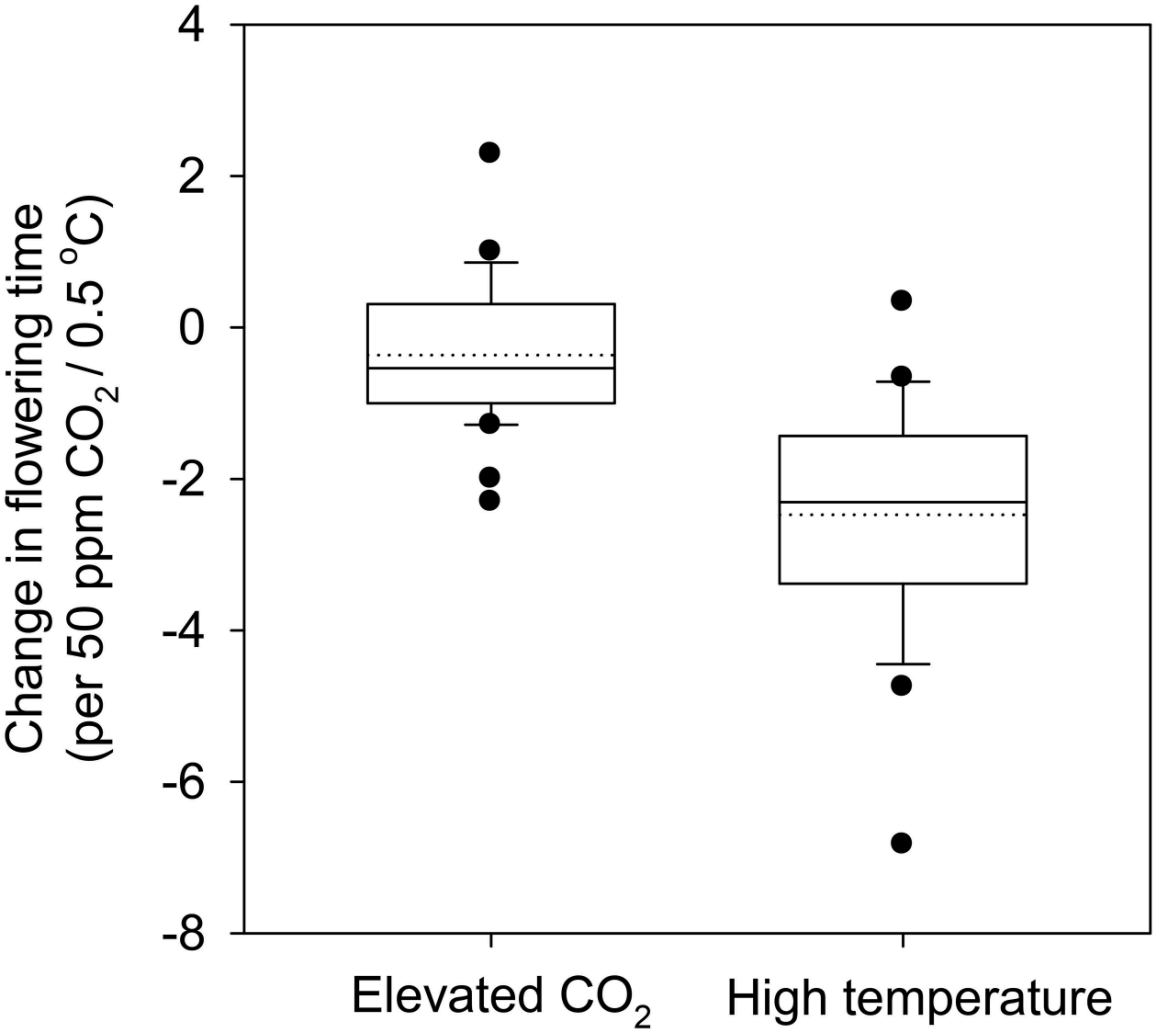
**Change in flowering time (days) in plants under elevated CO_2_ (per 50 ppm) and high temperature (per 0.5°C)**. The difference in CO_2_ concentration (elevated vs. ambient) was divided by 50 ppm to get a numerical unit for the total change in CO_2_ levels. Change in flowering time (days) was then divided by the previously obtained numerical unit to get change per 50 ppm. Similarly, change in temperature (high vs. ambient) was divided by 0.5 to get a numerical unit and change in flowering time (days) was divided by respective numerical unit associated with studies referenced in Table 1 and Table S1. The bottom and the top of the box represent the 25th and 75th percentile while the band near the middle represents the 50th percentile. The whiskers represent the 5th and the 95th percentile and the dots the outliers. References provided in Table 1 and Table S1.

Temperature affects flowering time by both affecting the rate of development directly and influencing vernalization (Craufurd and Wheeler, 2009). Vernalization is defined as the requirement for a period of chilling in winter to either break dormancy (in trees) or as an essential prerequisite to respond to other flowering-promoting stimuli in winter crops such as winter wheat or barley, and many bulb species (Amasino and Michaels, 2010). Predicted increase in mean temperatures of up to 3.7°C by the end of the century (IPCC, 2013) could have a significant impact on vernalization. For instance, a global analysis of the change in the “safe-winter-chill” period under different climate change scenarios suggests that there is likely to be a significant reduction in safe-winter-chill in warmer regions, which could have severe impacts on flowering time and production of temperate fruit and nut trees (Luedeling et al., 2011). With temperatures projected to increase at a higher rate during winters than during summers in cold regions, experimentally increased winter warming (0.4–2.4°C) resulted in a significant reduction in flower number and seed production. Interestingly, the impact of warmer winter temperatures was greatest on multi-inflorescence species rather than the single inflorescence species (Liu et al., 2012). Historical flowering date data of more than 400 plant species and deciduous trees at decadal time scale over a few centuries provides convincing evidence that flowering times have advanced by 4–6 days per single degree centigrade increase on average (Figure 1; Table 1; Table S1). For example, observations on flowering time in plant communities in Concord, Massachusetts, USA collected from 1852 through 2006 (Rushing and Primack, 2008), wherein temperature increased by 2.4°C, led to 7 days advancement in flowering date. Field-based experiments with annual crop plants using [CO_2_] and supplementary heating (temperature free-air-controlled enhancement (T-FACE); White et al., 2011), allowing wheat plants to be exposed to temperatures ranging from <0°C to >40°C, resulted in significantly early heading with increasing temperature (for details, see Figure 2 in White et al., 2011).

**Table 1:** **Range in flowering time variation (days) under elevated CO_2_ and warming temperature at species to ecosystem level**.

**Group/Species**	**Delay (+) or advancement (−) in flowering time**			
	**e[CO_2_]**	**Warming**	**References**	**Duration^#^**
Annual grasses	0 to +6	−2 to −4	Cleland et al., 2006	2000–2002
Annual forb	−2 to −4	−2 to −3	Cleland et al., 2006	2000–2002
Biennial forb	+4	+1	Cleland et al., 2006	2000-2002
*Phlox drummondii* (annual)	−2 to −4	−	Garbutt and Bazzaz, 1984	1980–1982
Temperate grassland	0	−3 to −19	Hovenden et al., 2008	2004–2007
*Asteraceae* (Herb)	−4	−	Johnston and Reekie, 2008	2004
Upland grassland ecosystem	0	−11 to −15	Bloor et al., 2010	2006–2007
Alien and native species of British Isles	−	−6 to −8	Hulme, 2011	1970–2000
Grassland nectar plants	−8 to +2	−	Rusterholz and Erhardt, 1998	NA^*^
C4 weeds	−11 to −15	−	Potvin and Strain, 1985	NA
Annuals	−9 to +16	−	Garbutt et al., 1990; Reekie and Bazzaz, 1991; Leishman et al., 1999	1984; 1994; NA
Desert shrubs	−	−20 to −41	Bowers, 2007	1894–2004
North American spring flowering species	−	−7	Miller-Rushing and Primack, 2008	1852–1858, 1878, 1888–1902, 2003–2006
Boreal trees	−	−3 to −11	Linkosalo et al., 2009	1846–2005
405 flowering plant species, UK	−	−2 to −13	Amano et al., 2010	1753–2003
Spring wheat	−1 to −3	−	Marc and Gifford, 1984	NA
Sunflower	−1 to −3	−	Marc and Gifford, 1984	NA
Spring wheat	−	−7 to −18	Olesen et al., 2012	1985–2009
Winter wheat	−	−4 to −14	Olesen et al., 2012	1985–2009
Spring oat	−	−6 to −17	Olesen et al., 2012	1985–2009
Maize	−	−3 to −21	Olesen et al., 2012	1985–2009
Soybean (*Glycine max*)	−2 to +11	−	Heinemann et al., 2006; Bunce, 2015	NA; 2013
Pigeon pea (*Cajanus cajan*)	+8 to +9	−	Sreeharsha et al., 2015	2012–2013
Rice	−7	−	Seneweera et al., 1994	NA
*Jatropa curcas* (Biofuel)	−8 to 10	−	Kumar et al., 2014	2011–2013

*Information on the type of individual species is provided in Table S1*.

# Duration of experiment conducted/duration for which data adopted for the analysis

* NA, not available

### Impact of e[CO_2_]

Flowering time in 40 published studies involving both crops and other plant species exposed to e[CO_2_] (from 350 to 1000 ppm) showed 28 cases (different species within the same study is considered a case) in which flowering time was earlier (average 8.6 days) and 12 cases in which flowering was delayed (average 5.2 days) (Figure 1; Table 1; see Table S1 for references and species). The effect of e[CO_2_] (680 ppm) on a grassland ecosystem using a FACE facility in California resulted in forbs flowering 2–4 days earlier, while in the dominant grass community flowering time was delayed by 2–6 days (Cleland et al., 2007). Reproductive traits in non-crop and wild species are known to respond less to e[CO_2_] than in crop species (Reekie et al., 1994; Jablonski et al., 2002; Kimball et al., 2002; Ainsworth and Long, 2004; see Table S1). Contrasting results have been documented showing no effect of e[CO_2_] on grass species in a mini-FACE experiment (Hovenden et al., 2008; Bloor et al., 2010) and similarly with maple trees (Norby et al., 2003). These differential responses could lead to changes in relative flowering times between species, thus affecting the ecosystem (Davis et al., 2010; Hulme, 2011). However, “Phenological complementarity” at an ecosystem level is known to promote coexistence of multiple species (McKane et al., 1990). Multiple environmental changes may alter the flowering time differentially in a natural landscape but the same would be more limited due to the domestication and breeding for uniformity in phenology among the crop plants.

In general, e[CO_2_] favors higher photosynthate (sugars and starch) accumulation in plants (Grodzinski et al., 1998; Springer and Ward, 2007). A sugar signaling metabolite trehalose-6-phosphate (T6P) showed a strong correlation (*r*^2^ = 0.94) with vegetative and shoot-apical meristem tissue sucrose levels in *Arabidopsis* (Wahl et al., 2013). T6P has been suggested to relay information about tissue carbohydrate availability and act as key signal for floral induction (Wahl et al., 2013). However, in *Arabidopsis thaliana*, the effect of e[CO_2_] on flowering time is the net result of the positive effect of e[CO_2_] on growth and a negative effect resulting from its tendency to increase leaf number, diluting the carbohydrate concentration in leaves at flowering (Johnston and Reekie, 2008). Interestingly, in *A. thaliana*, exposure to e[CO_2_] significantly advanced flowering time within a generation; however, across 15 generations, e[CO_2_] did not advance flowering time to the same level in each succeeding generation (Teng et al., 2009). Although the research was conducted in controlled environments, it provides clues that plants grown at e[CO_2_] for short-term may not evolve specific adaptations to e[CO_2_]. This was further confirmed by a reciprocal sowing experiment, which showed that e[CO_2_] did not produce detectable maternal effects on the offspring after 15 generations, indicating that e[CO_2_] may generate immediate change *via* phenotypic plasticity, but fail to produce genetic change (Teng et al., 2009).

Besides variable responses of flowering time under e[CO_2_] across non-crop species, studies with agricultural crops have shown an overall positive impact of e[CO_2_] on growth and yield. Most of this positive effect was attributed to a longer vegetative phase due to delays in flowering time under e[CO_2_], e.g., in pigeon pea (Sreeharsha et al., 2015), soybean (Bunce, 2015) and rice (Shimono et al., 2009). Flowering time is particularly sensitive to a variety of biotic and abiotic stresses that are expected to become more prevalent under future climates. Crops (or specific cultivars) with more flexibility to adjust flowering time to ensure vulnerable development phases escape stress under adverse environmental conditions could well determine their successful adaptation (Kazan and Lyons, 2016). Thus, molecular mechanisms regulating flowering time under a combination of e[CO_2_] with different biotic and abiotic stresses would help in tailoring climate resilient crops and utilize additional carbon for increasing yields under future climate.

### Interaction effects of climate change factors

Since e[CO_2_] would invariably drive temperatures higher, and with most climatic factors operating in tandem under field conditions, their combined effect on flowering time needs systematic evaluation. With a 4°C increase in temperature, flowering time was advanced by 50 and 31 days for *Chenopodium album* and *Setaria viridis*, respectively; but in combination with [CO_2_](1.8 times ambient [CO_2_]) it was 47 and 32 days, respectively (Lee, 2011). Moreover, with 22 different *Asteraceae* species, it was observed that e[CO_2_] advanced flowering by 4 days and in combination with increased temperature the flowering was advanced by an additional 3 days (Johnston and Reekie, 2008). Conversely, in several grass species it was demonstrated that e[CO_2_] delayed flowering by 2–7 days and high temperature (1.5°C above ambient) accelerated flowering by 2–5 days; in combination elevated [CO_2_] completely overcame the accelerated flowering time observed with increased temperature, i.e., a zero net change (Cleland et al., 2006). In another experiment the flowering and fruiting time of a grassland community with warming (increase in temperature +4.17°C), doubled precipitation and warming plus doubled precipitation (+4.83°C) was tested for one season (Sherry et al., 2007). Doubled precipitation had small and inconsistent effects whereas the warming plus doubled precipitation treatment was similar to warming only. This was to be expected as mean temperature varied by only 0.67°C with double precipitation and water-deficit stress has to be extreme to influence phenology (Craufurd et al., 1993). In summary, given that a doubling of [CO_2_] is predicted to occur by the end of the century, the observed changes in flowering time of 4–6 days or slightly more across a wide range of species are fairly modest, that is, <1 day per decade at most. It can be concluded that temperature is the major determining factor altering flowering time and ecosystem dynamics (see also Hovenden et al., 2008). There are contrasting responses between crop and non-crop or wild species, which may be associated with: (i) a combination of external factors such as elevated [CO_2_], nitrogen levels (Cleland et al., 2007); and (ii) due to the balance between “phenological complementarity” and resource acquisition, which further reduces the impact observed by e[CO_2_]. However, interactions between e[CO_2_] and temperature or photoperiod on flowering time can't be generalized and may be specific to a localized environment or plant species. Crops response to combined elevated CO_2_ and high temperature has been primarily quantified using controlled environment chambers (Madan et al., 2012) or poly tunnels (Dias de Oliveira et al., 2013, 2015a,b), wherein other interacting environmental factors such as wind speed, radiation could be considerably different compared to field conditions (Bahuguna et al., 2015). Developing economically feasible facilities to test this combined environmental change on field crops has been a major challenge and hence there is limited information on crops responses to this interaction under field conditions.

### Molecular mechanisms of flowering time regulation

Flowering time is governed by a complex signaling network involving the regulation of environmental stimuli (i.e., photoperiod, temperature) as well as endogenous genetic traits. Until now, four major flowering pathways have been identified. The vernalization and photoperiod pathways mediate signals from the environment, the autonomous pathway monitors endogenous signals based on developmental state of the plants, and the gibberellic acid (GA) pathway forms the fourth distinctive promotive pathway (for details see Amasino, 2010; Amasino and Michaels, 2010). Although there are increasing number of studies elucidating the gene network regulating flowering, information on the impact of climate change factors (temperature, e[CO_2_]) on the regulation of flowering time pathways is limited.

Genetic screens have identified a group of late-flowering mutants of *Arabidopsis* representing the photoperiod, gibberellin and autonomous pathways. Flowering time in these mutants is affected by change in ambient temperature (16-23°C) via a thermo-sensory pathway that mediates flowering regulation through the autonomous pathway genes *FCA* and *FVE* via a *FLOWERING LOCUS C* (*FLC*)-independent pathway (Blázquez et al., 2003). Conversely, some microRNAs (miRNAs are non-coding RNAs that negatively regulate the expression of their target genes) are reported to regulate flowering time in plants under ambient temperature (Lee et al., 2010; Kim et al., 2011). Lee et al. (2010) showed that loss of *SHORT VEGETATIVE PHASE* (*SVP*) function alters the miR172 expression level, suggesting that *SVP* is an upstream mediator of miR172. Hence, it is hypothesized that *SVP*-miR172 signaling is ultimately integrated by *FLOWERING LOCUS T* (*FT)*. This hypothesis is supported by early flowering of the miR172-overexpressing plants at high and optimum temperature (23 and 16°C, respectively) through up-regulation of *FT* expression independent of *SVP*. The SVP−miR172 regulatory circuit is proposed as a fine-tuning mechanism in response to changes in ambient temperature, which allows plants to modify their development toward changing temperature conditions (Lee et al., 2010). Likewise, the role of miR399 and its target gene *PHOSPHATE 2 (PHO2)* in flowering time regulation has been highlighted with miR399b over-expression and a loss of function allele of *PHO2* resulting in early flowering under higher temperature (23°C) and long day conditions in *Arabidopsis* (Kim et al., 2011). The authors suggested that miR399-*PHO2* module maintains phosphate homeostasis and regulates flowering time with increased expression of *TWINSISTER OF FT* (*TSF*). However, an indirect consequence of phosphate toxicity in miR399 overexpressing-*pho2* mutant plants could also lead to early flowering and warrants further investigation (Kim et al., 2011; for review see Spanudakis and Jackson, 2014).

**Figure 2:**
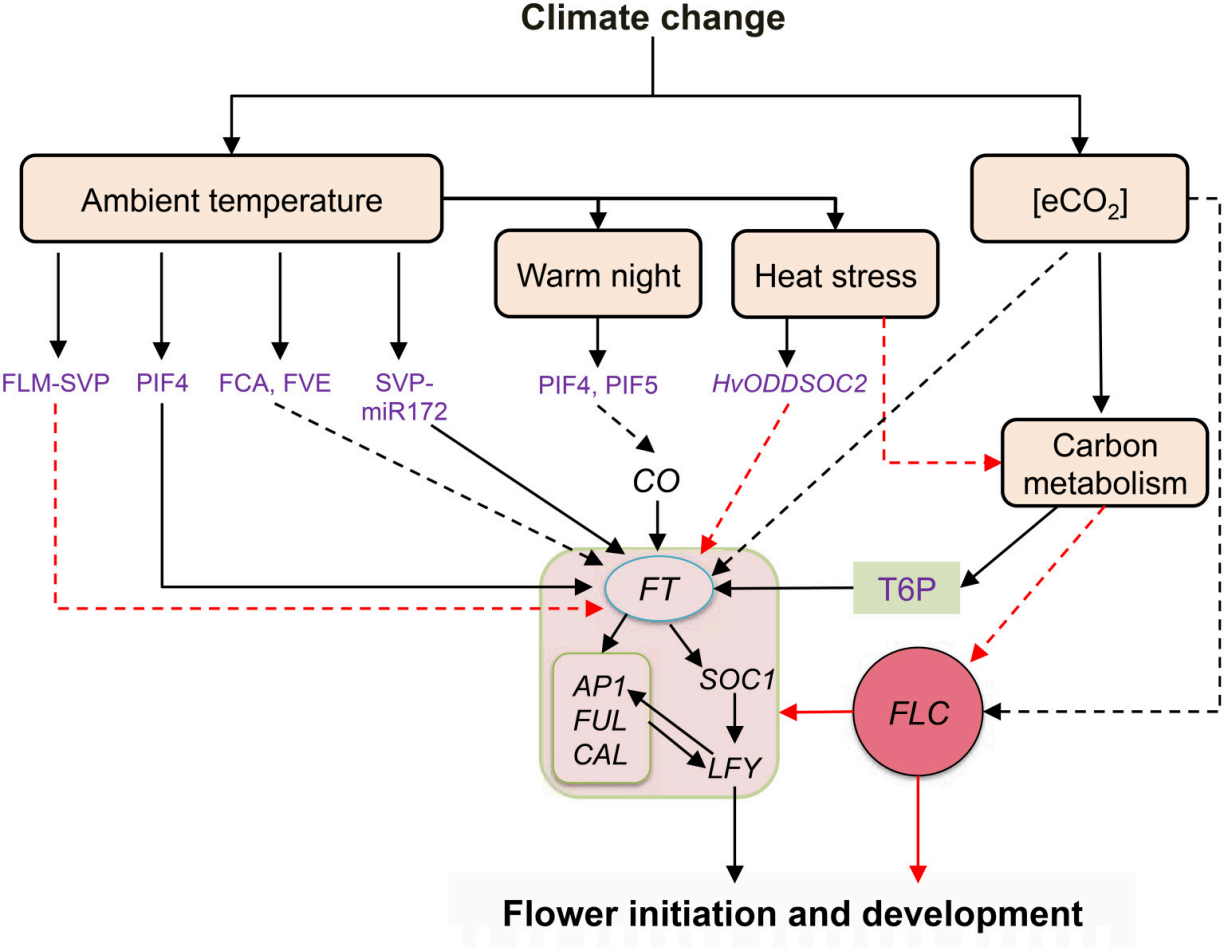
**Summary of flowering regulation by ambient temperature and e[CO_**2**_]**. Solid black and red arrows represent definite positive and negative interaction, respectively whereas broken black and red arrows depict plausible positive and negative interactions, respectively. Ambient temperature regulates *FT* expression by different mechanisms including PIF4 dependent and independent pathways. Warmer night temperature could induce early morning flowering by accumulating PIF4, PIF5 which regulate *CO* mediated *FT* expression. Heat stress may affect flowering events by hampering carbon metabolism and sugar signaling or by inducing floral repressor *HvODDSPC2*. Conversely, e[CO_2_] may directly regulate *FT* expression through floral repressor *FLC* or alternatively, positive impact of e[CO_2_] on carbon metabolism and sugar signaling may induce flowering pathway genes by suppressing *FLC* expression. Major flowering pathways genes affected by temperature and e[CO_2_] are represented in the box in the center. Abbreviations: *AP1, APETALA1*; *CO, CONSTANS*; *CAL, CAULIFLOWER; FLC, FLOWERING LOCUS C*; *FLM, FLOWERING LOCUS M; SVP, SHORT VEGETATIVE PHASE; FT, FLOWERING LOCUS T*; *FUL, FRUITFULL; HvODDSOC2, A MADS-box ßoral repressor; LFY, LEAFY*; *PIF4, PHYTOCHROME INTERACTING FACTOR 4*; *SOC1, SUPRESSION OF OVEREXPRESSION OF CONSTANS 1*; T6P, trehalose-6-phosphate.

A basic helix-loop-helix transcription factor, PHYTOCHROME INTERACTING FACTOR 4 (PIF4) activates *FT* gene expression in *Arabidopsis*. Binding of PIF4 at *FT* promoter is temperature-dependent, with an approximately five-fold increase in binding at high temperature (27°C) compared to ambient temperature (12°C) (Kumar and Wigge, 2010). H2A.Z-nucleosome occupancy declines at higher temperatures suggesting that the presence of H2A.Z-nucleosomes is limiting PIF4 binding to the promoter of *FT*. A possible epigenetic modification allows removal of H2A.Z from nucleosome to allow *PIF4* and *FT* transcription (Kumar et al., 2012). Interestingly, a recent report has documented that PIF5 along with PIF4 regulate *FT* expression under warm temperature (22°C) in *Arabidopsis*. Both PIF4 and PIF5 physically suppress phyB photoreceptor mediated suppression of *CO* resulting in early flowering under warm night conditions (Thines et al., 2014). Hence it can be concluded that warm night causes a phase shift in PIF4 and PIF5 accumulation in dark hours that favor early *FT* transcript increase during the daytime. Moreover, this pilot report indicates a possible mechanism of earlier TOA under warmer nights as documented in studies involving rice (Julia and Dingkuhn, 2013). Another temperature dependent mechanism of flowering regulation comprises two FLOWERING LOCUS M (FLM) protein splice variants (FLM-β and FLM-δ) which compete for interaction with the floral repressor *SVP* to regulate flowering (Pose et al., 2013). The predominant form of *SVP*-*FLM*-β complex at low temperatures prevents precocious flowering. The competing *SVP*- *FLM*-δ complex is impaired in DNA binding and acts as a dominant negative activator of flowering at higher temperatures. Apart from this, it can also be proposed that the opposing activities of two different splice variants of *FLM* and alternate splicing of transcription factors (which are temperature dependent), constitute a different regulatory pathway that acts in parallel to *PIF4* to support the transition from vegetative to reproductive development with a change in ambient temperature (Figure 2).

In Chinese narcissus, *Narcissus tazetta* var. chinensis, extended exposure to constant heat treatment (30°C) compared with low (15°C) and natural (ranging between 19 and 33°C) conditions promoted flower initiation and the *Arabidopsis FT* homolog *FT*-*like* (*Narcissus FLOWERING LOCUS T1*) *NFT1* gene was up-regulated under higher temperature exposure (Li et al., 2013a). However, in chrysanthemums, reduced expression of floral inducer *FLOWERING LOCUS T-like 3* (*FTL3*) coincided with delayed flowering as a consequence of high temperature (30°C) compared with control (20°C) treatment (Nakano et al., 2013). On the other hand, Hemming et al. (2012) reported progressive reproductive development in *Triticum aestivum* and *Hordeum vulgare* at higher temperature (25°C) under long days, whereas, under short days, the expression of *FT*-like genes was not responsive to 25°C heat treatment. A MADS-box floral repressor *HvODDSOC2* that is highly expressed at high temperature in short days might contribute to the inhibition of early reproductive development, suggesting close interaction between high temperature and day-length conditions. These studies suggest that, under higher temperature, the expression of *FT/FT*-like genes might be species-/crop-specific and flowering induction might share common and distinct factors with those of the photoperiodic flowering pathway.

Effect of e[CO_2_] on flowering time has been reviewed by Springer and Ward (2007) who summarized 60 studies including 90 different crop and wild species grown under e[CO_2_] in controlled chambers and the field (using open top chambers and FACE) conditions. The physiological basis of e[CO_2_] effect on flowering time was to enhance relative growth rates, increase plant size at flowering and raise tissue sugar status (Springer and Ward, 2007). Contrasting responses of flowering time to e[CO_2_] among short and long day plants suggested a possible interaction of the photoperiod pathway with e[CO_2_] to regulate floral signaling (Reekie et al., 1994). In *Arabidopsis*, the sustained expression of the floral repressor gene *FLC* was reported to be associated with delayed flowering in the genotype that was selected for high seed yield under e[CO_2_] (Springer et al., 2008; Figure 2). Recently, *MOTHER OF FT AND TFL1* (*MFT*), a homolog of *FT* and *TERMINAL FLOWER 1* (*TFL1*), has been identified as a candidate gene influencing flowering time with e[CO_2_] (Ward et al., 2012). In comparison to the impact of temperature, the current literature on the involvement of e[CO_2_] influencing the expression of flowering genes is limited. Only recently, the involvement of sugars in the regulation of flowering has been reported in *Arabidopsis*, wherein adequate sugar levels in the vegetative tissue (leaf) and shoot apical meristem produce TREHALOSE-6-PHOSPHATE (T-6-P) as a proxy signal for floral transition and initiation under inductive environmental conditions (Wahl et al., 2013). Interestingly, *Arabidopsis* plants with mutation in the *TREHALOSE-6-PHOSPHATE SYNTHASE* gene (AT1G78580) failed to flower, showing the essential role of sugar signaling (*T-6-P*) in regulation of flowering time (vanDijken et al., 2004). Elevated [CO_2_] can induce floral transition with enhanced substrate supply through increased photosynthesis (Grodzinski et al., 1998; Springer and Ward, 2007; Figure 2). However, excess foliar sugar accumulation (beyond a threshold) under e[CO_2_] may delay flowering in several plant species (for review see Springer and Ward, 2007). For instance, e[CO_2_] delayed flowering in *Arabidopsis* plants with a 41 and 105% increase in foliar sucrose and starch content, respectively (Bae and Sicher, 2004), indicating differential response to foliar sugars levels below and above threshold limits. Thus, varying sensitivity to sugar concentration under e[CO_2_] within or across species warrants further investigation to find a links between e[CO_2_] and flowering competency. It is envisioned, in the near future, that the yet-to-be-identified novel regulators involved in the signaling network modulating floral initiation in response to elevated temperature and e[CO_2_] will facilitate understanding and identifying options to develop plants or breed crops to better adapt to changing climate.

### QTLs and natural variation

It is essential to identify contributing candidates (either QTLs or candidate genes) influencing flowering time at the genetic level to accurately estimate phenological shifts or to select crops better adapted to changing climates. To map regions responsible for e[CO_2_] responsiveness, a random inbred line (RIL) population between the *Arabidopsis* cultivars Cvi-0 (no response) and SG (delayed flowering time), was studied and a major QTL contributing 30% of the variation in flowering time was mapped on Chromosome 1 (Ward et al., 2012). Subsequent mutant analysis resulted in the identification of *MFT* as a candidate gene for altered flowering time under elevated [CO_2_] of 700 ppm (Ward et al., 2012).

Genetic analysis of high temperature responsiveness in grapevines was studied using a bi-parental population following a growing degree day approach. This resulted in the identification of two independent QTLs responsible for variation in phenological duration to flowering. By utilizing the complete grapevine genome sequence, a number of candidate genes influencing flowering were identified including *FRUITFUL, SEPALLATA*, and *FLC*. However, *VvFT* on chromosome 7 and a *CONSTANS*-like *VvCOL2* gene on chromosome 14 were the most reliable candidates identified from two of the underlying QTLs co-localizing for the flowering time (Duchêne et al., 2012). Over expression of *VvFT* in *Arabidopsis* is shown to hasten flowering (Carmona et al., 2007) while *VvCOL2* is known to be associated with genetic variations of flowering time in *Medicago truncatula* (Pierre et al., 2010) and *Medicago sativa* (Herrmann et al., 2010). By employing the diverse global core collection of 473 *Arabidopsis* accessions and subjecting them to four simulated climatic conditions representing current and future climatic scenarios (2010, 2025, 2040, and 2055), a genome wide association analysis (GWAS) identified five independent main-effect QTLs for both days to flowering and thermal sensitivity (Li et al., 2013b). A total of just 12 and 7% of the phenotypic variation was accounted by all the five candidates SNP locations identified for flowering time and thermal sensitivity, respectively, with the polygenic background accounting for 76% of the variation. Further, a lack of association between candidate SNPs for both the above traits failed to help explain their role in the *Arabidopsis* distribution across the latitudinal gradient. The need to engage in more local populations to estimate the actual contributions of key genomic regions to flowering time with lesser polygenic interference is proposed. On the other hand, using 950 diverse rice accessions, classified into rice *indica* and *japonics* sub-species, it was concluded that a larger sample increased the power of detecting traits including flowering time through GWAS analysis (Huang et al., 2012). The lower phenotypic variation observed by Li et al. (2013b) could be explained by either the alteration in flowering time with increased temperature or too small population. Conversely, two independent studies have exploited the early TOA trait from *O. rufipogon* (Thanh et al., 2010) and *O. officinalis* (Ishimaru et al., 2010) and stable QTLs on chromosome 4, 5, 10 and 3, 8, respectively have been identified. The lack of co-localizing of identified QTLs indicates the role of environmental factors such as radiation, photoperiod and relative humidity, indicating a strong genotype x environment interaction as proposed by Kobayashi et al. (2010). Phenotyping for these traits in breeding programs needs to be rigorous in order to avoid or minimize artifacts of these environment interactions.

In conclusion, temperature is by far the most important climate change factor affecting flowering time, with warmer temperatures predominantly advancing flowering. The molecular pathways and the genes involved in flowering time under optimal conditions are well documented. However, information on their response to high temperature and interactions with other environmental factors such as e[CO_2_], water-deficit stress and photoperiod, is limited and warrants detailed investigation. More studies under realistic field conditions, such as in FACE and T-FACE experiments, are needed.

## Future perspectives

Some of the key knowledge gaps and opportunities to overcome adverse impacts of climate change on flowering time and processes include:

1. Precise and accurate predictions of changes in phenology, flowering time and time of day of flowering (TOA) using crop simulation models can be attained by: (i) employing canopy/floral tissue temperature rather than ambient air temperature, (ii) incorporating the differential impact of day and night temperature amplitude, and (iii) considering the key interactions between environmental factors such as temperature and RH (VPD).
2. Genetic outputs obtained using global core accessions have and will result in identifying genomic regions (QTLs/SNPs/candidate genes) responsible for changes in flowering time using experimental and natural temperature regimes. The applicability of the candidates identified needs validation at smaller spatial scale to demonstrate the advantage in overcoming yield losses caused due to changes in the timing of flowering related events.
3. Traits such as early-morning flowering, as demonstrated in rice, need to be explored in other crop species or plants as well. A more careful examination and documentation of TOA across landraces and wild relatives of crops could provide novel and pragmatic solutions for adapting crop production to rising temperatures.
4. Deciphering differential regulation of flowering genes by TFs such as *bHLH PIFs* (*PIF3, PIF4, PIF5*) and microRNAs that might be involved in integrating diverse environmental signals (light, temperature and possibly CO_2_) to regulate flower initiation and development is needed. Additionally, novel unknown regulators of flowering and their interconnections or independencies with other already known pathways under changing climates constitute future direction of research on flowering time response to environmental stimuli.

## Author contributions

All authors listed, have made substantial, direct and intellectual contribution to the work, and approved it for publication.

### Conflict of interest statement

The authors declare that the research was conducted in the absence of any commercial or financial relationships that could be construed as a potential conflict of interest.
